# Fertility treatment using assisted reproductive technology during immune checkpoint inhibition: report of two cases

**DOI:** 10.1093/oncolo/oyag273

**Published:** 2026-07-15

**Authors:** Keri Tomechko, Tali Azenkot, Deirdre A Conway, Sandip Pravin Patel, H Irene Su

**Affiliations:** School of Medicine, University of California San Diego, San Diego, CA, 92093, United States; Moores Cancer Center, University of California, San Diego, CA, 92037, United States; Utah Fertility Center, Utah, United States; Moores Cancer Center, University of California, San Diego, CA, 92037, United States; Moores Cancer Center, University of California, San Diego, CA, 92037, United States; Obstetrics, Gynecology, and Reproductive Sciences, University of California, San Diego, CA, United States

**Keywords:** immune checkpoint inhibitor, immune-related adverse event, solid tumor, survivorship, oncofertility

## Abstract

There are limited data to guide fertility counseling for patients on prolonged immune checkpoint inhibition. Concerns include potential teratogenicity, decline in ovarian reserve and spermatogenesis, and endocrine immune-related adverse events such as hypophysitis. As indications for checkpoint inhibition expand, an increasing number of reproductive-aged patients face fertility-related decisions around active therapy. We present two patient cases, one male and one female, who used assisted reproductive technology to support family building during maintenance immune checkpoint inhibitor therapy. A female lung cancer survivor underwent in vitro fertilization and froze six euploid blastocyst embryos for gestational surrogacy. A male gastroesophageal junction cancer survivor and his female partner underwent in vitro fertilization followed by a medicated intrauterine insemination cycle to conceive healthy fraternal twins. While prospective data are needed, this case series supports the feasibility of fertility treatment during checkpoint inhibitor therapy and highlights remaining clinical questions regarding this important aspect of cancer survivorship.

## Introduction

Immune checkpoint inhibitors (ICIs) have transformed the management of advanced malignancies, including those affecting reproductive-aged patients. For these young patients with potential for cure, family building is an important component of cancer care and survivorship.^1^ However, the feasibility of fertility treatments during active ICI exposure is not known.

For females, teratogenicity, prematurity, low birth weight, and fetal immune-related toxicities inform recommendations not to use ICIs during pregnancy (Table 1).^2^ In murine models, ICIs were shown to deplete ovarian follicular reserve and impair oocyte maturation, likely through T-cell infiltration and ovarian inflammation.^3^^,^^4^ While human data are limited, in a small cohort (*n* = 28), lower post-treatment ovarian reserve was observed after CTLA-4 inhibition.^5^

**Table 1.** Fertility considerations related to immune checkpoint inhibition in males, females, and fetuses.

Consideration	Male	Female	Fetal
**Immune-related adverse events**	Parental hypothyroidism, hyperthyroidism, hypophysitis, 1° adrenal insufficiency		Fetal enterocolitis, pneumonitis, endocrine abnormalities
**Direct gonadal effects**	Impaired spermatogenesis; testicular and ejaculate immune-cell infiltration; autoimmune orchitis	Possible decreased ovarian reserve (AMH decline); rare autoimmune oophoritis	Unknown
**Clinical and laboratory findings**	Reduced sperm count and motility	Irregular menses	Preterm birth, fetal growth restriction
**Fertility prior to cancer therapy**	Fertility counseling and fertility preservation referral; reliable contraception for pregnancy prevention		N/A
**Fertility during or after cancer therapy**	Fertility evaluation, including semen analysis; unassisted attempts if normal spermatogenesis; routine infertility treatment (inseminations and IVF) if abnormal	Fertility evaluation; autologous oocyte preservation via IVF and gestational surrogacy	N/A

Abbreviations: AMH, anti-Müllerian hormone; IVF, in vitro fertilization.

For males, ICIs have also been associated with potential adverse effects on fertility (Table 1). In murine models and clinical case series, ICIs have been shown to induce testicular immune cell infiltration, which may disrupt spermatogenesis.^6–8^ Inflammatory infiltrates in the ejaculate and abnormal semen parameters have also been documented.^6^^,^^7^ Pharmacovigilance analyses indicate that abnormal spermatogenesis is the male reproductive adverse event most strongly associated with ICIs, although the absolute number of reported cases is low.^9^

**Figure 1.**
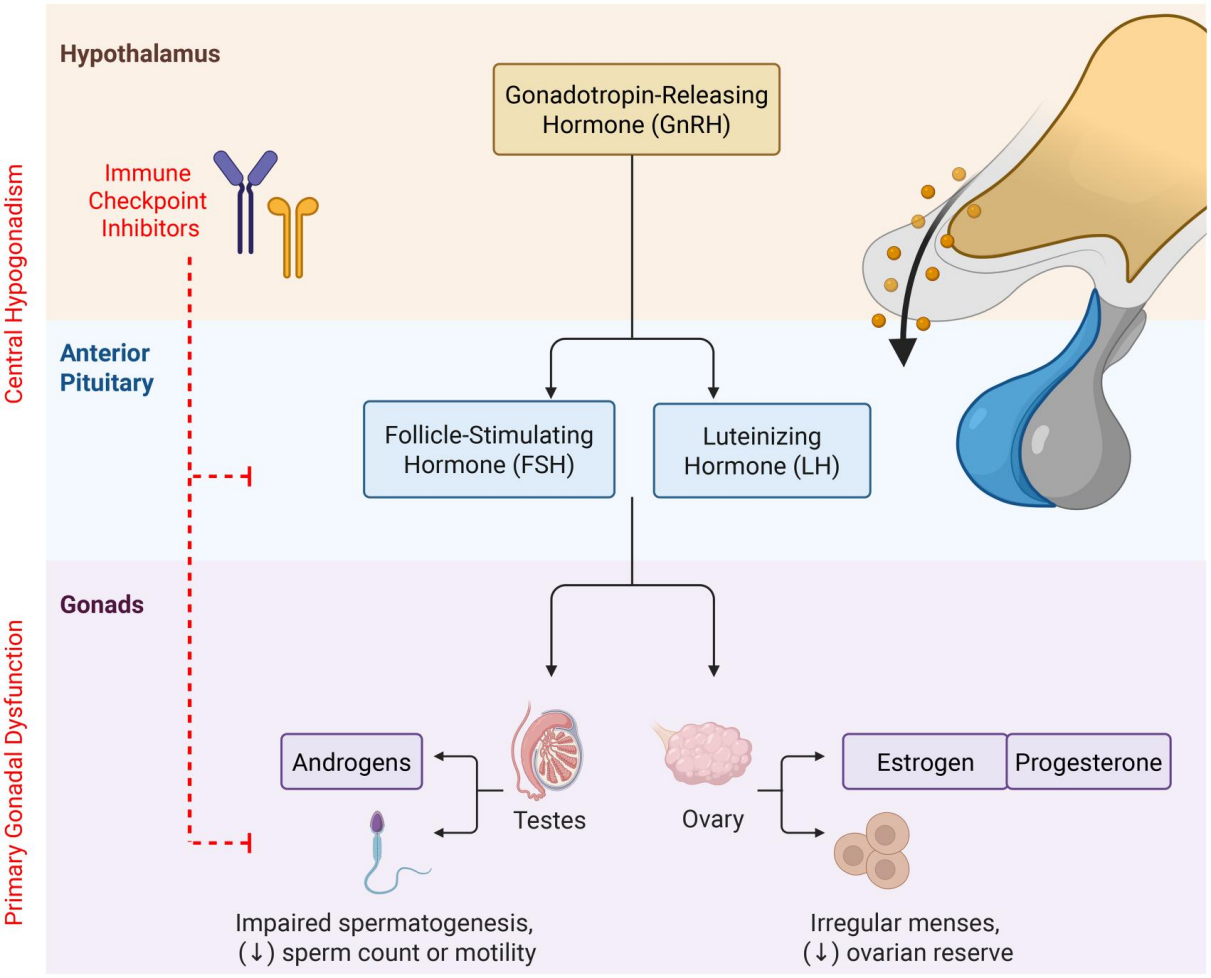
Mechanisms by which immune checkpoint inhibition may impact the hypothalamic-pituitary-gonadal axis. Solid arrows indicate physiologic signaling; dashed red lines indicate hypothesized immune-related adverse effects. Effects displayed may be transient or permanent. Created with BioRender.com.

For both sexes, immune-mediated endocrine toxicity may also impact fertility or pregnancy health.^2^ In persons treated with combination anti-CTLA-4/PD-1 therapy, hypophysitis and hypothyroidism are estimated to occur in 6.4% and 13.2% of patients, respectively (Figure 1).^10^ ICI-mediated thyrotoxicosis, primary adrenal insufficiency, and diabetes can also occur.

Aligning with these concerns, there is a paucity of clinical data describing fertility interventions in persons with ongoing ICI therapy. As individuals trying to conceive are excluded from most clinical trials, unique patient experiences offer practical insights. Here we present cases of a female and a male who pursued fertility using assisted reproductive technology (ART) during ICI therapy.

## Cases

### Case 1

A 33-year-old previously healthy female, gravida 1, para 1, never-smoker presented with a subacute cough. She was found to have a left lower lobe mass, with biopsy showing large cell carcinoma. She underwent lobectomy and diaphragm resection but developed widespread recurrence prior to adjuvant therapy. She began chemo-immunotherapy shortly after surgery with carboplatin, paclitaxel, ipilimumab, and nivolumab and achieved stable disease. Carboplatin and paclitaxel were discontinued after one cycle due to infusion reaction, and she continued maintenance ipilimumab/nivolumab. Prior to systemic therapy, she was counseled on fertility preservation via embryo banking but declined. She was treated with leuprolide during platinum-based chemotherapy. At 16 months post diagnosis, her imaging continued to show stable disease on maintenance ipilimumab and nivolumab; she and her male partner wished to have another child.

After extensive counseling, the patient elected to attempt embryo creation using ART without stopping ICI therapy, followed by gestational surrogacy. She underwent a GnRH antagonist cycle with luteal phase ovarian stimulation 15 months after initiating chemo-immunotherapy, approximately 14 months after discontinuation of carboplatin/paclitaxel, and 9 days after her most recent ICI dose. Baseline antral follicle count was 14 and starting gonadotropin doses were 150 units of follicle stimulating hormone and 75 units of human menopausal gonadotropin daily. Following 12 days of stimulation and peak estradiol 2717 pg/mL, 14 oocytes were retrieved; 13 were in metaphase II. Following intracytoplasmic sperm injection, all mature oocytes fertilized, and six blastocysts were cryopreserved. There were four euploid, one mosaic, and one aneuploid blastocysts. The couple has an ongoing clinical pregnancy via surrogacy, while the patient’s malignancy continues to be stable on ICI therapy. Notably, she was diagnosed with eosinophilic esophagitis, which may be related to ICI therapy and is well controlled on 2.5 mg of prednisone daily; she has not experienced any other immune-related adverse events.

### Case 2

A 41-year-old previously healthy man presented with refractory epigastric pain. Endoscopy demonstrated a large circumferential mass in the distal esophagus. Biopsy confirmed stage IIIC mucinous adenocarcinoma of the gastroesophageal junction. He underwent multimodality therapy including two combination chemotherapy regimens (4 cycles doxorubicin, cyclophosphamide, and docetaxel, then 5 cycles leucovorin, fluorouracil, and oxaliplatin), followed by concurrent chemoradiation. He was started on adjuvant pembrolizumab shortly after surgery with subsequent sustained complete response. He did not experience any immune-related adverse events. Prior to systemic therapy, he was counseled on fertility preservation and completed sperm banking; however, his specimen was reported as lost by the cryopreservation facility.

With continued complete response on pembrolizumab, the patient and his 41-year-old wife desired pregnancy but did not wish to stop ICI therapy. The patient remained on pembrolizumab throughout semen analysis, IVF, and intrauterine insemination. More than 2 years after his last chemotherapy exposure, the patient underwent semen analysis following 6 months of unprotected intercourse without conception, which demonstrated oligoasthenospermia (volume 2.4 mL; concentration 3 × 10^6^/mL; motility 10%; total motile count 0.7 × 10^6^). The couple underwent IVF approximately 1 year later, by which time semen parameters had normalized. The IVF cycle was unsuccessful as only one embryo grew to the blastocyst stage and was aneuploid. The couple then pursued ovulation induction with letrozole and intrauterine insemination. The insemination sample demonstrated normal parameters (volume 3 mL; concentration 120 million/mL; total motile count 45 million); it was purified using a density gradient processed through centrifugation, then resuspended and re-centrifuged to remove any remaining debris. After this first intrauterine insemination cycle, the couple conceived fraternal twins, delivered via cesarean section without congenital anomalies. One twin was diagnosed with autism spectrum disorder; the patient has a family history of this condition.

## Discussion

The cases presented illustrate the intersection of immunotherapy and fertility care, a growing challenge as ICIs are increasingly used in reproductive-aged patients. For females receiving ongoing ICI therapy, fertility using autologous oocytes is feasible via IVF and gestational surrogacy or can be delayed until treatment cessation. In our first case, we observed encouraging outcomes following a standard IVF protocol and timing for retrieval. While we considered stopping ICI, the long half-life (15–28 days) would have required at least 5 months off cancer-directed therapy prior to IVF, which was considered unacceptable for cancer control.^10^

Male patients should also be counseled on fertility risks and preservation with sperm and/or embryo banking before cancer treatment.^1^ Chemotherapy remains the primary driver of gonadotoxicity, though ICIs may also disrupt spermatogenesis.^8^ Our second case demonstrates that family building can be achieved during ICI exposure using standard treatments for infertility. The couple’s cause of infertility was likely multifactorial, including initial male factor from decreased spermatogenesis that recovered over time (likely related to chemotherapy rather than ICI), as well as female age. Although the largest pharmacovigilance study of ICI-exposed pregnancies did not identify overreporting of adverse fetal or neonatal outcomes, rare reported complications following in utero ICI exposure include prematurity, transient congenital hypothyroidism, and immune-related neonatal toxicities; however, longitudinal studies are needed to investigate associations with offspring health, including neurocognitive disorders.^11^

## Conclusion

Taken together, the reported cases demonstrate the preliminary feasibility of family building during ICI therapy. This experience offers oncology and fertility clinicians information on fertility options for a growing number of patients with cancer treated with checkpoint blockade. We await data from prospective observational trials to further inform this clinical dilemma.

## Data Availability

De-identified data related to this case are available from the corresponding author upon reasonable request.
